# Advanced age woman with diminished ovarian reserve obtained live birth following a zero pronuclei-derived four-cell frozen-thawed embryo transfer on day 4: a case report

**DOI:** 10.3389/fmed.2024.1462425

**Published:** 2024-09-24

**Authors:** Xiao-lei Zhang, Yong-qian Chen, Ya-juan Zhang

**Affiliations:** Department of Reproductive Medicine, The First Affiliated Hospital of Harbin Medical University, Harbin, China

**Keywords:** advanced age woman, diminished ovarian reserve, frozen-thawed, slow-growing embryo, live birth

## Abstract

**Objective:**

Advanced maternal age and diminished ovarian reserve (DOR) are challenges in infertile patients for *in vitro* fertilization-embryo transfer (IVF-ET). This study aimed to investigate the pregnancy outcomes of women with advanced age and DOR undergoing low-quality embryo transfers.

**Case report:**

We report a rare case of successful pregnancy resulting from a zero pronuclei (0PN)-derived four-cell embryo transfer on day 4 (D4). An advanced age patient with DOR underwent five unsuccessful embryo transfers. A successful outcome was achieved when the patient underwent a hormone replacement FET cycle and received 0PN-derived four-cell frozen-thawed cleavage embryo transfer on D4. Fourteen days after the transfer, her serum β-human chorionic gonadotropin level was 575.3 mIU/mL. Subsequent prenatal examinations were normal, and the patient delivered a full-term healthy baby girl by caesarean section. Achieving a successful pregnancy after 0PN-derived four-cell frozen-thawed embryo transfer on D4 is rare. This increasingly exhibits significance for advanced age patients with DOR.

**Conclusion:**

Selectively transferring embryos with slow growth but low fragments and no evident damage is beneficial for advanced age patients with DOR. For these individuals, obtaining embryos is challenging. Therefore, a personalized embryo transfer strategy should be considered to increase the possibility of pregnancy.

## Introduction

Several advanced women (>35 years old) with diminished ovarian reserve (DOR) require *in vitro* fertilization and embryo transfer (IVF-ET). Owing to the decrease in oocyte quantity and decline in oocyte quality, obtaining oocytes from these patients during controlled ovarian stimulation is challenging, which further complicates acquiring high-quality embryos (1). Many DOR patients require multiple consecutive oocyte retrievals, and the choice of ovarian stimulation protocol differs from that in women with good ovarian reserve. After achieving the desired number of frozen embryos, frozen-thawed embryo transfer (FET) can be performed.

Research showed that embryos that continued to grow after being thawed had better developmental potential (2). Moreover, embryo development was more synchronised with endometrial growth, increasing the possibility of pregnancy. Blastomere growth is observed through overnight culture to evaluate the developmental potential of thawed embryos. Retrospective studies have found that the pregnancy rate was most optimal if the number of blastomeres doubled after overnight culture of thawed embryos. Embryos with an increased number of blastomeres had a higher pregnancy rate than those without an increased number of blastomeres (3). Some slow-developing thawed embryos could still continue to grow and achieve pregnancy after overnight culture. Currently, cleavage-stage embryos on day 3 (D3) are the primary choice for freezing. Well-developed embryos should grow to 6–8 cells or more on D3. An embryo that only developed to four cells on D3 was of lower quality. If it remained at four cells after overnight culture to day 4 (D4), it showed very poor developmental potential, and clinicians commonly decided to discard it rather than transfer. Reports of successful pregnancy after a four-cell embryo transfer on D4 are few.

In IVF-ET, the fertilization status of oocytes is assessed based on the presence of pronuclei. The normal fertilization of an oocyte is identified when two distinct pronuclei (2PN) and two polar bodies (2PB) are observed within 16–20 h post-insemination. Oocytes showing zero pronuclei (0PN) are considered abnormally fertilized (4). On post-insemination day 1, mature oocytes with 0PN do not undergo division and remain unfertilized, referred to as 0PN oocytes, or they can continue to develop to the first division, termed 0PN-derived embryos. Studies have found that 0PN-derived embryos have a low pregnancy rate and should be discarded. However, other researchers who conducted extensive cytogenetic analysis on embryos derived from 0PN found that some of these embryos are diploid, and an adequate percentage of 0PN embryos can develop into high-quality blastocysts (5, 6). This indicates that mature oocytes with 0PN during fertilization may be fertilized zygotes with 2PN. Early pronuclei breakdown was considered a good indicator of embryo viability (7). The primary reason for not observing 2PN could be that the fixed time for fertilization check misses the actual fertilization time for 0PN-derived embryos (8).

In the present case, the patient with DOR never experienced embryo implantation despite multiple embryo transfers. Only one 0PN-derived four-cell frozen embryo on D3 remained. After thawing, the embryo showed no cell damage. However, after overnight culturing to D4, the embryo did not grow and remained at four blastomeres. With no other embryo options available, the patient underwent a transfer with this embryo, resulting in pregnancy and delivery. Currently, her daughter is over 4 years old and in good health.

## Case presentation

The patient first visited our hospital on 15 February 2017, at the age of 37 years; her husband was 39 years old. The couple had previously undergone multiple unsuccessful IVF-ET treatments at other hospitals. She experienced menarche at the age of 13, with a regular menstrual cycle of 25 days, lasting 3–4 days each cycle. She does not experience dysmenorrhoea and has no family history of DOR or other diseases. She denied having a history of hypertension, diabetes, thyroid diseases and other underlying conditions. Her karyotype is 46,XX,1qh+ with standard G-banding chromosome (at 320–400 band level). Her BMI was 24.6 kg/m^2^.

In 2004–2005, the patient became pregnant twice but had elective abortions in the early stage of pregnancy because of personal reasons. Subsequently, she used an intrauterine device (IUD) for contraception for 5 years. In 2010, she had the IUD removed in an attempt to conceive. After trying to conceive for 1 year without success, she underwent multiple fallopian tube flushing treatments, which also proved unsuccessful. In 2013, she was diagnosed with bilateral tubal hydrosalpinx and underwent laparoscopic bilateral tubal ligation. Between 2014 and 2015, she sought IVF-ET treatment at another hospital and was diagnosed with DOR. Within 2 years, oocyte retrieval was performed three times, with standard IVF each time. However, all three embryo transfers failed to result in implantation. In 2016, an ultrasound examination revealed a pelvic encapsulated effusion, for which she underwent an aspiration procedure. The result of the T-cell test for tuberculosis was negative.

In 2017, the patient opted for further IVF-ET treatment in our hospital due to fallopian tubal issue. An initial examination showed AMH <0.06 ng/mL and two sinus follicles in both ovaries, demonstrating severely DOR. Antagonist protocol was used for ovarian stimulation in the first IVF-ET cycle, retrieving one MII oocyte. Standard IVF was performed, and one 2PN-derived three-cell embryo on day 2 was obtained. No pregnancy was achieved after the transfer. After a 7-month break, the patient underwent the second IVF-ET cycle. The ovarian stimulation protocol was changed to a mild stimulation approach utilizing Clomiphene Citrate (Fertilan, Codal Synto Ltd.) and Human menopausal gonadotropin (HMG, Livzon), and one MII oocyte was retrieved. Standard IVF was conducted, and one 0PN-derived four-cell embryo on D3 was obtained. In this cycle, the patient’s endometrial thickness was 5 mm; hence, the embryo was cryopreserved. To date, the patient has undergone four unsuccessful embryo transfers. Only one poor-quality four-cell embryo derived from 0PN was saved. Considering the extremely low ovarian reserve of the patient, oocyte retrieval was recommended to accumulate embryos and avoid oocyte exhaustion in the ovaries. Following a 3-month break, the patient underwent a third IVF-ET cycle. Given the poor embryo quality observed with the mild stimulation protocol and the presence of a thin endometrium, we opted for the antagonist protocol for repeat ovarian stimulation. Two MII oocytes were successfully retrieved. Post-standard IVF, a 2PN-derived five-cell embryo and a 2PN-derived four-cell embryo were observed on D3, both of which exhibited slow growth. To monitor their ongoing developmental potential, culture was extended to D4. The two embryos progressed to eight-and seven-cell stages, respectively. In this cycle, the endometrial thickness was 9 mm. The transfer of both embryos was decided on; however, implantation was unsuccessful.

**Figure 1:**
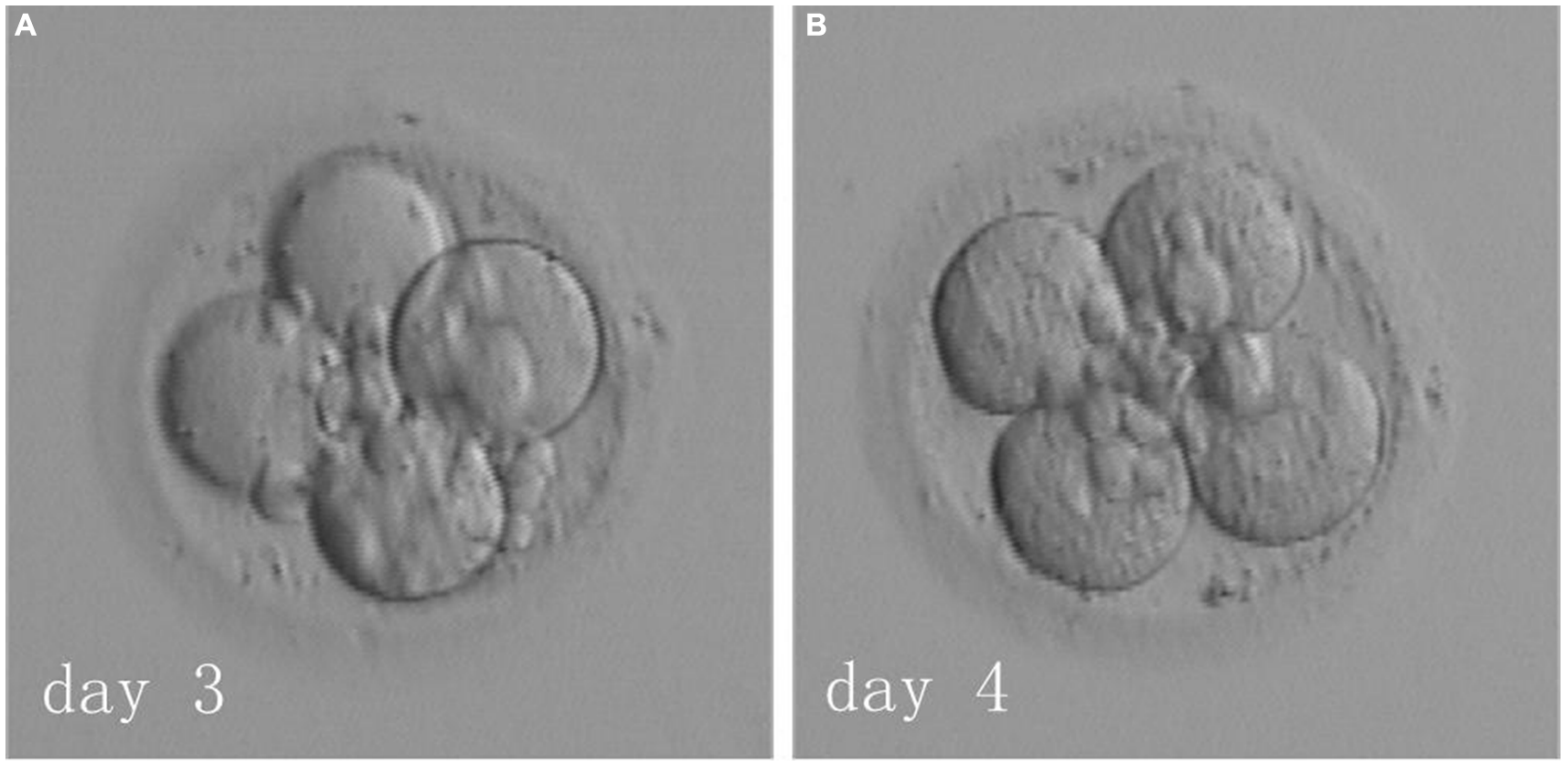
Embryo on day 3 **(A)** and on day 4 **(B)**.

The patient underwent six IVF-ET cycles involving oocyte retrieval across two hospitals and five subsequent fresh transfers, none of which resulted in a successful pregnancy. The only remaining embryo was a four-cell embryo derived from 0PN on D3. The couple decided against further oocyte retrieval and opted to have this singular embryo thawed and transferred. Should this attempt prove unsuccessful, they resolved to discontinue treatment. In June 2018, the FET cycle was performed using a hormone replacement endometrial preparation. On day 3 of her menstrual cycle, the patient started on a daily dose of 4 mg of 17β-estradiol (Femoston, Abbott biologicals B.V.). By day 6, a daily regimen of 75 mg aspirin was introduced. On day 15, the patient’s endometrial thickness was 9 mm, and hormone levels were as follows: estradiol (E_2_), 314 pg/mL; progesterone, 0.2 ng/mL; and luteinizing hormone, 11.39 mIU/mL. The patient was administered a daily injection of 40 mg of progesterone (Progesterone Injection, Zhejiang Xianju Pharmaceutical Co., Ltd.) and 30 mg of dydrogesterone (Duphaston, Abbott Biologicals B.V.) to facilitate endometrial transformation. On day 3 of transformation, the 0PN-derived four-cell embryo on D3 was thawed (Figure 1, left side) and cultured overnight. By day 4, it was observed that the embryo had not grown further and still consisted of four cells (Figure 1, right side), and the couple chose to have the embryo transferred. On day 14 following transfer, the patient’s blood test showed a β-human chorionic gonadotropin level of 575.3 mIU/mL. Further, on day 33, ultrasound indicated a single intrauterine pregnancy with normal fetal bud and cardiovascular pulsation. At 39 weeks of pregnancy, a 3,400 g and 50 cm healthy baby girl was delivered via caesarean section, with no observed abnormalities until the present.

## Discussion

Successful pregnancy and subsequent childbirth of the patient are notably uncommon in the case report. Some factors are unfavourable for the success of IVF-ET. First, when the patient presented to our department, she was 37 years old, placing her in the advanced maternal age category. It is medically accepted that advanced maternal age patients have a lower pregnancy rate with IVF-ET compared with younger individuals. Second, the patient had AMH <0.06 ng/mL and few sinus follicles in both ovaries, indicating an extremely diminished ovarian reserve. During ovarian stimulation, retrieving a sufficient number of oocytes in advanced age women can be challenging. This is crucial in determining whether they can successfully obtain embryos and achieve pregnancy. The patient previously underwent five embryo transfers at two hospitals, none of which resulted in implantation. Patients with multiple implantation failures typically have a lower success rate in subsequent IVF-ET. Under such a difficult situation, the patient reluctantly decided to have one 0PN-derived four-cell embryo transferred on D4. Nevertheless, she successfully conceived and delivered a healthy girl. This outcome is notably rare in clinical cases.

Chromosomal polymorphisms are believed to not transmit genetic information or induce phenotypic abnormalities, thereby having limited clinical and pathological relevance. However, clinical observations have shown that chromosomal polymorphisms can be associated with recurrent spontaneous abortion, fetal abnormalities, diminished sperm quality and infertility. The patient’s chromosomal karyotype is 46,XX,1qh+, signifying an enhancement in the secondary constriction (qh+) on the long arm. This is a prevalent type of chromosomal polymorphic alteration. Secondary constrictions are primarily located in the heterochromatin regions of the long arms on chromosomes 1, 9 and 16. Changes in these constrictions result from the addition or removal of highly repetitive DNA sequences. The patient’s embryos were not cultured to the blastocyst stage, and no chromosomal testing was performed on the embryos. Therefore, whether the poor embryo quality was associated with her chromosomal karyotype was not analysed.

Oocytes that are normally fertilized experience pronuclear fading approximately 23–25 h after fertilization (9, 10). During this time, it is difficult to ascertain whether the observed 0PN status is from normal fertilization or remains unfertilized. Studies utilizing time-lapse monitoring for pronuclear assessment showed that the incidence rate of embryos derived from 0PN was significantly lower than with fixed-time pronuclear assessment methods. Furthermore, embryos with earlier pronuclear fading tended to develop more rapidly. 0PN-derived blastocysts could result in the birth of healthy babies (11). Thus, if embryos assessed as 0PN at a fixed time can continue to divide, they may be those developing at a faster pace, with their pronuclei already disappearing by the time of fertilization observation. The use of time-lapse imaging is beneficial in reducing 0PN occurrences. Our department conventionally uses the fixed-time pronuclear assessment method, potentially missing pronuclei observation. This can result in a certain proportion of embryos being inaccurately classified as derived from 0PN. In this case, the embryo derived from 0PN ultimately achieved pregnancy, indicating that it was possibly of 2PN origin. Owing to the strict observation timing, the emergence of the pronuclei was not detected, leading to its misidentification as an embryo from 0PN. However, this embryo demonstrated slower development, contrary to the faster growth observed in other studies.

It is preferable for an embryo to develop to 6–8 cells or more within 3 days of culture. Although the embryo transferred in the present case displayed slower development, it had few cellular fragments. According to our laboratory protocols, four-cell embryos derived from 0PN with a low fragment rate can be cryopreserved. Consequently, we presented the patient with the choice of either continuing embryo culture for blastocyst or immediate cryopreservation of the embryo. Recent studies have also demonstrated that the transfer of blastocysts derived from 0PN can result in subsequent favorable pregnancy and neonatal outcomes (12, 13). The patient opted for embryo cryopreservation directly instead of blastocyst culture. After thawing, embryos of poor quality were cultured overnight to further assess their developmental potential. If the number of blastomeres increases by at least one after overnight culture, such an embryo is considered to have the capability to continue development and can be transferred. After thawing, the patient’s embryo exhibited no signs of damage. Following overnight culture, the embryo remained to have four blastomeres. Based on our standard protocol, this embryo should be disposed of. However, for this advanced age woman with DOR, while respecting her wishes, we further determined that 0PN-derived four-cell frozen-thawed embryo on D4 has the potential for pregnancy and delivery.

The patient’s previous attempts at embryo transfer involved fresh embryos, all of which yielded unsuccessful outcomes. However, the last transfer involved a frozen embryo and resulted in a successful delivery. FET may enhance endometrial preparation and synchronization with the embryo, thereby increasing the likelihood of success for patients with diminished ovarian reserve (DOR). Further investigation is needed to explore whether FET can improve pregnancy outcomes following hormonal replacement cycles.

The transfer of such slow-growing embryos to patients has not been conducted in our department of reproductive medicine for nearly 30 years, and no similar clinical cases have been found in the databases on line. Therefore, we believe that this is a unique case, even if it may not be applicable to every patient. For advanced age patients with DOR, obtaining embryos becomes more difficult. Consequently, whether the quality standards for embryos suitable for transfer should be expanded needs to be discussed. When advanced age patients with DOR lack available embryos for selection, an individualized embryo transfer strategy should be considered rather than disposing of all embryos. We recommend that embryos with slower growth but minimal fragments and no obvious damage post-thawing can be selectively transferred to enhance the possibility of achieving pregnancy.

## Data Availability

The original contributions presented in the study are included in the article/supplementary material, further inquiries can be directed to the corresponding author.
